# Analysis of Developmental Level of Counties of Fars in Terms of Health Infrastructure Indicators Using Standardized Scores Pattern Approach and Factor Analysis

**DOI:** 10.5539/gjhs.v7n1p240

**Published:** 2014-09-25

**Authors:** Hassan Zahmatkesh, Aziz Rezapoor, Farshad Faghisolouk, Amir Hossein Eskandari, Amin Akbari, Mehdi Raadabadi

**Affiliations:** 1 >Hospital Management Research Center, Iran University of Medical Sciences, Tehran, Iran; 2 Department of Health Economics, School of Health Management and Information Sciences, Iran University of Medical Sciences, Tehran, Iran; 3 Health Management and Economics Research Center, Iran University of medical sciences, Tehran, Iran; 4 Department of Health Management and Economics, School of Public Health, Tehran University of Medical Sciences, Tehran, Iran; 5 Ministry of Health and Medical Education, Tehran, Iran; 6 Research Center for Health Services Management, Institute for Futures Studies in Health, Kerman University of Medical Sciences, Kerman, Iran; 7 Students Scientific Research Center (SSRC), Tehran University of Medical Sciences, Tehran, Iran

**Keywords:** development, standardized score, factor analysis, Fars

## Abstract

**Introduction:**

It is necessary for planning in order to achieve optimal development, to have knowledge and understanding of the current situation. This identification requires separate areas of study into planning and assessing regions of each area with development indicators and analysis and ranking each area in terms of having gifts of development. The study also aims to analyze the development level of counties of Fars in terms of health infrastructure indicators using standardized scores pattern and factor analysis.

**Methods:**

This is a descriptive and applied study, which has discussed the levels of 29 counties of Fars based on 10 health selected indicators using a standardized scoring model. Data were collected using a data collection form developed by the researchers through the Center of Statistics and Shiraz University of Medical Sciences. Results were analyzed using Excel and SPSS 19.

**Results:**

Based on calculations according to standardized score and factor analysis methods, Shiraz and Rostam had the most and the least level between the other cities, respectively. Also development coefficient and operating score of the studied counties ranged from a maximum of 0.894 to a minimum of -0.941, and a maximum of 3.861 to a minimum of 2.001, respectively.

**Discussion:**

There are relatively large differences between different counties in healthcare sector, and most studied counties in terms of healthcare sector indicators are not satisfactory. So planning how to allocate healthcare resources from policy makers to improve the studied counties is essential.

## 1. Introduction

Planning social services, formulating development strategies and success implementing projects, identification and evaluation of capabilities, deficiencies, and determining developmental levels of regions based on a set of appropriate indicators is inevitable. Thereby managers in executive levels are allowed to specify development strategies based on the specific requirements of each region and provide programs coordinated and appropriate to regional conditions (Taghvaei & Avargani, 2007). The planning aims to achieve optimal development and establish balance, which primarily need to understand and identify the status quo. This identification requires separate areas of study into planning and assessing regions of each area with development indicators and analysis and ranking each area in terms of having gifts of development (Zarabi, Mohammadi, & Rakhshaninasab, 2008). Inequities and its dimensions are symptoms of underdevelopment, because actually those countries are known as developed countries that in addition to high social and economic indicators, their income distribution is relatively equitable in its communities, while these values are lower and also its distribution is very unfair (Dadashpour, Alizadeh, & Madani, 2011). In order to assess the level of development, GDP and counties’ per capita were first compared and ranked. Assessing development through this method due to taking into account the amount of equity in the distribution of educational services, health care, etc was limited (Mahmoudi, 2011). Several indicators are considered to assess the level of development in a temporal and spatial range, and health is one of these indicators (Amanpoor, Esmaeily, & Jokar, 2012). Among the various development indicators, healthcare index due to its great role in ensuring public health is one of the most important indicators of progress in any country and the success of national development plans to a large extent depends on achieving the goals of this section. Whatever amount and quality of health indicators in a community is more and their distribution is more balanced and appropriate, relative prosperity and more health will exist in that society (Nastaran, 2001). Health and its establishment on basic principles of development are inevitable. Having the required health care needs in Iran has been known as the most fundamental public rights and Article 29 of the Constitution has explicitly emphasized on it. In this regard, the formal sector of health in order to secure and promote the physical, psychological and social health level of the society based on defined policies is a systematic set of activities and executive operations (Mansour, 2004). A glance at the health indicators in the country in the last decade shows on one hand the rapid improvement of the indicators and other on the other hand some inequality in some indicators in different regions and provinces of the country (Movahedi et al., 2009). In any case, it is necessary that Iran like any other developing country pay special attention to the health sector development in order to improve its development position among countries in the world because development in this sector is a prerequisite for the development of other sectors of society. Without a healthy society and people with physical, psychological and social health, it is pointless to address development in other sectors. In order to plan development in health sector of a society, it is first necessary to examine its status in terms of having health indicators (Yang, Zhang, Jia, & Ci, 2005). Today, the issue of development is a concern for many countries. In other words, the development is anything but making a more satisfying life condition for public (Coovadia, Jewkes, Barron, Sanders, & McIntyre). In terms of classifying and developing health in country several studies have been conducted in large scales in counties by mathematical models. Seyedin et al. (2013) in a study entitled “Classification of Kermanshah Province Districts in Terms of Health Structural Indicators Using Scalogram Model” examined the status of the structural health indicators in three groups of institutional, manpower and rural health indicators, and concluded that there is a large gap in terms of having structural health care indicators among different counties of Kermanshah (Mousavi et al., 2013). Amini et al. (2006) in a research using a combination method of factor analysis and taxonomic analysis investigated health ranking of different provinces of country based on having 35 development indicators in health field. The results showed that Isfahan, Tehran, and Markazi were in good health status, but Ardabil, Golestan, and Qom had undesirable health level. In addition, the health status of Khuzestan, Sistan and Baluchistan, and Kohkilooyeh and Boyer Ahmad was in a critical condition (Amini, Yadollahi, & Inanlou, 2006). In other countries some studies have been performed as well, such as Soares et al. (Soares, Marquês, & Monteiro, 2003) in Portugal, Wulan and Petrovic (Wulan & Petrovic, 2012) and Yannis and Andriant (Phillis & Andriantiatsaholiniaina, 2001), which using factor analysis and cluster models and fuzzy logic performed classification of regions with the use of health and economic indicators. Despite all the criticism since 1970s raised on the use of quantitative models in urban issues, if mathematical models are formulated in simple forms and with a limited number of variables, they can help to get a clearer understanding of urban phenomena. One of the quantitative models in order to evaluate and rank the areas is standardized score models and factor analysis. Therefore in order to achieve this important issue and social justice, classification of areas and identifying their development degree in terms of health indicators and determining the capabilities and shortcomings seem essential. This study also aims to analyze the development level of counties of Fars in terms of health infrastructure indicators using standardized scores pattern and factor analysis.

## 2. Method

This is a descriptive and applied study. In this study, using standardized scoring model and factor analysis the health level of counties of Fars has been investigated based on health indicators in 2011. Therefore, the geographic scope of the study is Fars and its statistical population consists of its 29 counties (Abadeh, Arsanjan, Estahban, Eqlid, Bavanat, Pasargad, Jahrom, Kharameh, Khorrambid, Khonj, Darab, Rostam, Zarrin Dasht, Sepidan, Sarvestan, Shiraz, Farashband, Fasa, Firuzabad, Qir and Karzin, Kazerun, Kavar, Gerash, Larestan, Lamerd, Marvdasht, Mamasani, Mohr, and Neyriz). After literature review (Bahadori, Shams, Sadeghifar, Hamouzadeh, & Nejati, 2012; Hamouzadeh, Moradi Hovasin, Sadeghifar, & To fighi, 2013; Movahedi et al., 2009; Nezafat, Hashjin, & Mehr, n.d.; Sadeghi Ravesh, Ahmadi, Zehtabian, & Tahmoures, 2013; Sheidai, Khatamsaz, & Mosallanejad, 2000; Taghvaei & Avargani, 2007) and expert opinions (To collect the useful and deep information and so selected best indicator, 8 managers and faculty, as the key informants in field of study, were selected using purposeful sampling method), 10 indicators were considered as selected health indicators including active medical institutions to thousand populations ratio, number of beds in active medical institutions to thousand populations ratio, healthcare institutions to thousand populations ratio, public healthcare institutions to active healthcare institutions ratio, daily healthcare institutions to active healthcare institutions ratio, circadian healthcare institutions to active healthcare institutions ratio, the number of laboratories to thousand populations ratio, the number of pharmacies to thousand populations ratio, radiology centers to thousand populations ratio, and rehabilitation centers to thousand populations ratio. Data were collected using a data collection form developed by the researchers including questions on the counties’ names, number of active medical institutions, available beds, number of healthcare institutions, number of public healthcare institutions, number of daily healthcare institutions, number of circadian healthcare institutions, numbers of laboratories, number of pharmacies, radiology centers, rehabilitation centers, and city population. Data were also collected by the Center of Statistics and Shiraz University of Medical Sciences. After completing the forms, the rank of city development was calculated using factor analysis and standardized scores through Excel 2010 and do SPSS.19, respectively. The method of calculation in these two methods is as follows: First, indicators are standardized in terms of the county based on standard score model.

Standardized scoring method is used to compare indicators and to obtain a single index from combined results of indicators. In fact, this standard scoring method is capable to reveal significant differences between regions in terms of defined indicators (Kazemi Mohammadi, 2001).


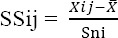


where,

SSij= Standardized score of index I for city j

Xij= value of index i for city j

xݲ= the indicators mean

Sni= Standard deviation of the index i

In next level the standardized scores of each indicators studied in each county are added together and the result is divided by the total number of indicators. The obtained score is the average standard score or development index of each county that provides a comparison in terms of development status as a single index:


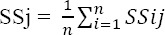


SSj= index for city j

N= number of indicators considered (Biranvand Zadeh, Sorkh Kamal, Alizadeh, & Sheykh Eslami, 2007).

Factor analysis method is also a developed multivariate statistical technique performed to reduce and restructure the data. Using the correlation between data is the main foundation of this analysis based on which many variables can be grouped (Laskowski et al., 2010).

Factor analysis, which is a multivariate statistical technique, aims to summarize the data and tries to justify correlation patterns in the distribution of a random vector in terms of the minimum number of unobservable random variables called factors. This method investigates internal correlation of a large number of variables and finally classifies and explains them in forms of limited factors. Stages of its implementation are as follows:

a) Selecting the appropriate variables
b) Extracting factors
c) Determining variables of each factor (interpreting the factor matrix) (Menke, 2012).

To determine the developmental gap in healthcare sectors among counties, five categories were considered including developed, moderately developed, averagely developed, less developed and undeveloped categories. After this stage, to determine the distances between the provinces in the five-level first the change in score range using “a” formula was obtained, then using “b” formula the distance between the categories was calculated, resulting in categorization of counties in five groups.


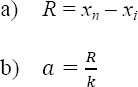


## 3. Results

Fars has a total population of 4596658, most of which live in Shiraz (1700687) and least of which live in Pasargad (31504). This province also has 68 active medical institutions, 7521 beds, 382 public healthcare institutions, 345 daily healthcare institutions, 184 circadian healthcare institutions, 396 laboratories, 549 pharmacies, 177 radiology centers, and 456 rehabilitation centers. Distribution of each variable is shown in Table 1.

**Table 1:** Distribution of health variables in the studied counties

County	Population	Active medical institutions	Available beds	Health care centers	Public health care institutions	Daily health care centers	circadian healthcare institutions	Laboratories	Pharmacy	Radiography centers	Rehabilitation centers
Abadeh	98188	1	127	11	9	7	4	11	11	4	9
Arsanjan	41476	1	32	5	5	5	0	4	4	1	1
Estahban	66172	1	93	9	7	5	4	6	6	1	4
Eqlid	93975	1	86	14	13	10	4	10	11	2	5
Bavanat	48416	1	32	9	9	8	1	5	4	2	1
Pasargad	31504	1	32	3	3	3	0	4	4	1	1
Jahrom	209312	2	391	32	30	28	4	15	22	8	17
Kharameh	61580	1	32	6	6	6	0	2	0	1	1
Khorrambid	50252	1	28	6	5	4	2	3	7	1	2
Khonj	41133	1	43	6	6	6	0	3	4	1	2
Darab	189345	1	140	19	16	14	6	10	14	3	7
Rostam	46851	0	0	6	6	5	1	0	1	0	0
Zarrin Dasht	69438	1	46	5	5	2	3	4	4	2	2
Sepidan	89398	1	51	16	15	14	2	9	9	2	2
Sarvestan	40531	1	25	4	4	4	0	1	0	1	1
Shiraz	1700687	35	4840	167	64	73	94	180	298	104	309
Farashband	42760	0	0	5	4	1	4	2	3	1	1
Fasa	203129	2	374	40	35	31	9	19	24	8	14
Firuzabad	119721	1	122	11	8	7	4	5	10	3	6
Qir and Karzin	65045	1	35	8	7	5	3	6	5	2	2
Kazerun	254704	1	163	25	22	18	7	16	20	4	12
Kavar	77836	0	0	10	7	6	4	3	2	1	2
Gerash	47055	1	117	4	4	4	0	2	3	1	3
Larestan	226879	3	198	21	19	15	6	21	19	4	19
Lamerd	83916	2	79	12	10	9	3	7	7	5	2
Marvdasht	307492	1	195	33	25	22	11	17	28	6	16
Mamasani	116386	1	119	19	17	16	3	14	15	3	8
Mohr	59727	1	25	9	8	7	2	8	4	2	1
Neyriz	113750	1	96	14	13	10	4	9	10	3	6
Total	4596658	65	7521	529	382	345	184	396	549	177	456

Source: Statistical Center of Iran, and Shiraz University of Medical Sciences

According to calculations done by the standard score method, the development coefficient of each county has been calculated. The results showed that the rate of development for the studied counties ranged from a maximum of 0.894 to a minimum of -0.941, so that Shiraz had the most and Rostam had the least rate among the others (Table 2).

**Table 2:** Coefficient and rank of development in studied county according to standardized score

Rank	County	Development coefficient	Rank	County	Development coefficient
1	Shiraz	0.894	16	Larestan	-0.01
2	Fasa	0.571	17	Neyriz	-0.019
3	Sepidan	0.531	18	Khorrambid	-0.026
4	Pasargad	0.478	19	Qir and Karzin	-0.078
5	Jahrom	0.43	20	Zarrin Dasht	-0.254
6	Bavanat	0.397	21	Sarvestan	-0.351
7	Mamasani	0.355	22	Firuzabad	-0.37
8	Abadeh	0.335	23	Kazerun	-0.382
9	Lamerd	0.331	24	Marvdasht	-0.404
10	Khonj	0.29	25	Darab	-0.442
11	Eqlid	0.199	26	Kharameh	-0.537
12	Arsanjan	0.171	27	Farashband	-0.613
13	Gerash	0.156	28	Kavar	-0.867
14	Mohr	0.114	29	Rostam	-0.941
15	Estahban	0.041			

Source: research calculations.

Based on a standardized scoring method according to the coefficients obtained, the studied counties were classified into 5 groups of developed, moderately developed, averagely developed, less developed, and undeveloped. A large number of counties (32%) were among the moderately developed and the least were among developed and underdeveloped groups (each 10%) (Table 3).

**Table 3:** Development level of counties of Fars in having health care indicators (standardized score)

Group	Class gap	Degree of having	Counties’ names	Number of counties	Percent
First	0.528-0.894	Developed	Shiraz, Fasa, Sepidan	3	10%
Second	0.161-0.527	moderately developed	Pasargad, Jahrom, Bavanat, Mamasani, Abadeh, Lamerd, Khonj, Eqlid, Arsanjan	9	32%
Third	-(0.206)-0.160	Averagely developed	Gerash, Mohr, Estahban, Larestan, Neyriz, Khorrambid, Qir and Karzin	7	24%
Fourth	-(0.573-0.207)	Less developed	Zarrin Dasht, Sarvestan, Firuzadab, Kazerun, Marvdasht, Darab, Kharameh	7	24%
Fifth	-(0.941-0.574)	Undeveloped	Farashband, Kavar, Rostam	3	10%

Source: research calculations.

Based on calculations carried out according to the factor analysis method, factor score of the counties studied ranged from a maximum of 3.861 to a minimum of 2.001. The same as standardized score method Shiraz had the most value and Rostam had the least value among other counties (Table 4).

**Table 4:** Score and rank of development of the studied counties based on factor analysis method

Rank	County	Factor score	Rank	County	Factor score
1	Shiraz	3.861	16	Eqlid	-0.188
2	Gerash	1.121	17	Qir and Karzin	-0.202
3	Abadeh	0.934	18	Marvdasht	-0.231
4	Jahrom	0.754	19	Zarrin Dasht	-0.302
5	Pasargad	0.645	20	Sarvestan	-0.357
6	Fasa	0.504	21	Darab	-0.422
7	Lamerd	0.471	22	Kazerun	-0.425
8	Larestan	0.299	23	Bavanat	-0.439
9	Estahban	0.262	24	Mohr	-0.561
10	Firuzabad	0.227	25	Sepidan	-0.681
11	Khonj	0.121	26	Kharameh	-0.819
12	Mamasani	0.080	27	Farashband	-1.146
13	Khorrambid	0.017	28	Kavar	-1.250
14	Arsanjan	-0.111	29	Rostam	-2.001
15	Neyriz	-0.160			

Source: research calculations.

## 4. Discussion

The first step to develop health sector and reduce the health gap among different regions is to achieve a relatively complete understanding of the health sector situation in those regions. Therefore, this study aimed to determine the level and extent of county development in the health sector in 2011 in Fars. In the model used the counties are ranked and also their development status is considered. To determine development level of the counties the standardized score model, five levels were considered including highly developed, developed, developing, poor and very poor.

Experience of the regional studies in different countries suggests that some areas compared to other areas of a country have a better development and growth. Therefore, if planners are able to identify factors affecting the development of the areas, then they can both benefit from the experiences of managers of different parts of the region and optimally allocate available funds (Girardi et al., 2012; Rosero-Bixby, 2004).

The results showed that in both methods Shiraz ranked first and Rostam ranked last in terms of development indicators for the healthcare indicators. The development coefficient variation in the standardized score method was 1.895 and in factor analysis was 5.862 indicating a large gap between the studied counties in terms of taking advantage of the health indicators.

Zarabi et al. (2008) developed a spatial analysis of development indicators of health care in Isfahan. Their study used 47 indicators in healthcare sector that showed their distribution was not balanced and there was a significant difference among the counties of Isfahan considering the development of healthcare services. Khansar and Borkhar and Meymeh were in highest and lowest level, respectively.

Bahadori et al. (2012) also performed classification of health structural indicators using Scalogram Model in Golestan Provonce, The results showed that there is a large gap in terms of taking advantage of structural health indicators among counties of Golestan. Aq Qala with 97 scores had the highest and Azad Shahr with 41 scores had the lowest level of utilization of structural health indicators.

Based on the standardized score model, 3 counties (10%) were in less developed level, 10 counties (34%) were underdeveloped, 7 (24%) were averagely developed, 12 (42%) were relatively developed and developed. Hamouzadeh et al. (2013) ranked West Azerbaijan districts regarding utilization of structural indices of health care using Scalogram Analysis Model. In their study evaluating the general situation of counties of West Azerbaijan Province regarding utilization of total structural indicators of health, three counties were determined as developed and highly utilized counties including Mahabad, Naqadeh, and Urmia. Khoy and Bukan were known as relatively developed counties. Takab, Maku, and Miandoab were considered as averagely developed, and less developed counties include Salmas, Shahindej, Sardasht, Oshnaviyeh, and Chaldoran.

Sayehmiri performed a sturdy in order to rank health status of the cities of Ilam using numerical taxonomy technique and principal component analysis and considering 66 major indicators of health. The results showed that Ilam was the most developed and Mehran, Darrehshahr, Dehloran, Shirvan-o-Chardavol, Eyvan, and Abdanan palced in the following ranks (Sayehmiri & Sayehmiri, 2001).

Therefore, the differences in the studied counties are largely due to the differences in access to health indicators. Although it was mentioned the differences between the different regions using different approaches also depend on the type of geographic area (socioeconomic development, etc), the distance to the provincial capital, the level in which the society is (levels of economic development and economic growth in the community), the performance of healthcare organizations in the region and confidence of local people to them, infrastructure facilities in the community, the extent of decentralization policies in the area, policies of workforce distribution utilities in university of medical sciences, and inability of the relevant units on competency and skills of staff. It can be also said that results of examining resource distribution status regarding the used model as a default in access and equity measurement in resource distribution can lead to development plans in this sector.

## 5. Conclusion

Health care indicators the same as other development indicators in third world countries have not been distributed in a balance form among geographical areas. Iran is no exception, and the gap is clearly seen in the development of these indicators in different provinces of Iran. In the present study, the rank and status of health sector development in provinces were examined.

The results show there is a relatively large difference among different counties in health sector, and most studied counties are not in a desirable level in terms of healthcare indicators. Only 10 percent of the counties were in developed status based on the model.

Generally, counties’ ranking in healthcare sector provides relevant authorities with more accurate planning, identifying strengths and weaknesses, and priority of resources in accordance with needs of the province. In summary, the results showed that statistical methods are effective tools in ranking and determining status of development in the healthcare sector. The results of this study regarding the allocation of resources for the health sector would be useful for health planners and policy makers.
